# Whole genome survey and microsatellite motif identification of *Artemia franciscana*

**DOI:** 10.1042/BSR20203868

**Published:** 2021-03-10

**Authors:** Euna Jo, Seung Jae Lee, Eunkyung Choi, Jinmu Kim, Sung Gu Lee, Jun Hyuck Lee, Jeong-Hoon Kim, Hyun Park

**Affiliations:** 1 Department of Biotechnology, College of Life Sciences and Biotechnology, Korea University, Seoul 02841, Korea; 2 Division of Life Sciences, Korea Polar Research Institute (KOPRI), Yeonsu-gu, Incheon 21990, Korea; 3 Department of Polar Sciences, University of Science and Technology, Yeonsu-gu, Incheon 21990, Korea

**Keywords:** Artemia franciscana, Genome assembly, Genome size, SSR

## Abstract

*Artemia* is an industrially important genus used in aquaculture as a nutritious diet for fish and as an aquatic model organism for toxicity tests. However, despite the significance of *Artemia*, genomic research remains incomplete and knowledge on its genomic characteristics is insufficient. In particular, *Artemia franciscana* of North America has been widely used in fisheries of other continents, resulting in invasion of native species. Therefore, studies on population genetics and molecular marker development as well as morphological analyses are required to investigate its population structure and to discriminate closely related species. Here, we used the Illumina Hi-Seq platform to estimate the genomic characteristics of *A. franciscana* through genome survey sequencing (GSS). Further, simple sequence repeat (SSR) loci were identified for microsatellite marker development. The predicted genome size was ∼867 Mb using K-mer (a sequence of k characters in a string) analysis (K = 17), and heterozygosity and duplication rates were 0.655 and 0.809%, respectively. A total of 421467 SSRs were identified from the genome survey assembly, most of which were dinucleotide motifs with a frequency of 77.22%. The present study will be a useful basis in genomic and genetic research for *A. franciscana*.

## Introduction

The genus *Artemia* (Crustacea: Branchiopoda: Anostraca), known as brine shrimp, is an aquatic invertebrate living mainly in salt lakes. To date, seven species have been assigned to the genus *Artemia* excluding the parthenogenetic populations called *Artemia parthenogenetica* [1]. *Artemia* species are important in aquaculture industry because their dormant cysts are easily hatched, and the nauplii can be used as a nutrient-rich food for fish [2,3]. In addition, they are also widely used as aquatic model organisms for ecotoxicity tests, along with *Daphnia* [4–6]. However, despite the significance of *Artemia* in aquaculture, genomic research is still incomplete, and the genomic characteristics are less known, compared with *Daphnia*, because of the relatively large estimated genome size of 0.93–2.93 Gb [7–9].

*Artemia franciscana*, a native species in North America, has been extensively used and introduced to other continents for commercial purposes, thereby affecting the local population’s biodiversity [10,11]. Additionally, there were some incorrect identifications, especially *A. franciscana* as *Artemia salina*, in citing the species used as test organisms in literature [12]. These problems, including population structure changes and species misidentification, suggest the need for not only morphological analyses but also population genetic studies and additional marker development to discriminate closely related species.

Genome survey sequencing (GSS) using next-generation sequencing (NGS) is a time- and cost-effective way to evaluate genome information, such as genome size, heterozygosity level, and repeat content, and can be used to develop molecular markers [13,14]. Simple sequence repeats (SSRs) or microsatellites are short tandem repeats of one to six nucleotides that have been utilized as genetic markers because of their outstanding abundance and high variability [15]. In *Artemia*, several microsatellite markers have already been developed for use in population genetic studies [16,17], but they are limited because they are not based on genome-wide data.

In the present study, we aimed to estimate the genomic characteristics of *A. franciscana* through GSS and then identify SSRs from GSS for microsatellite marker development. The present study would be useful for population genetics and molecular species identification and as a framework for subsequent whole-genome sequencing of *A. franciscana*.

## Materials and methods

### Materials and DNA extraction

Nauplii of *A. franciscana*, originating from the Great Salt Lake (Utah, U.S.A.), were hatched on commercial cysts (INVE Technologies NV, Dendermonde, Belgium). Cultures were maintained in 30 g/l salt water at 25°C with aeration. Live *Tetraselmis* sp. was fed to *A. franciscana* during the culture period. One egg-bearing female individual was cultured separately and the progenies were used in the subsequent experiments. Genomic DNA was extracted from whole five adults using phenol/chloroform method. The quality and quantity of the DNA were checked using a BioAnalyzer (Agilent Technologies, Santa Clara, CA, U.S.A.) and Qubit fluorometer (Invitrogen, Life Technologies, Carlsbad, CA, U.S.A.).

### Genome sequencing, assembly, and K-mer analysis

Genomic DNA was randomly sheared into 350-bp fragments using an ultrasonicator (Covaris, U.S.A.). A paired-end DNA library was prepared and sequenced with Illumina Hi-Seq 2000 platform. To ensure the quality of data, adaptors, poly(N) sequences, and low-quality reads were filtered out, and only clean reads were subjected to K-mer (a sequence of k characters in a string) analysis. K-mer analysis was performed using Jellyfish 2.1.4 [18] with a K-value of 17, 19 and 25. Based on the 17-mer distribution, GenomeScope [19] in R version 3.4.4 [20] was used to estimate genome size, heterozygosity rate, and repeat content. The *de novo* genome assembly was carried out using Maryland Super-Read Celera Assembler (MaSuRCA) version 3.3.4 [21].

### SSR detection and primer design

Genome-wide SSR identification was conducted using QDD version 3.1.2 pipeline [22]. First, the assembled sequences were used to extract microsatellite sequences with di- to hexanucleotides motifs. Next, sequences were compared using all-against-all BLAST to detect unique singleton sequences. In the last step, primer pairs were designed using two iterative methods for each sequence.

## Results and discussion

### Genome sequencing data statistics

In the present study, a total of 22.8 Gb of raw data for *A. franciscana* were generated by Illumina paired-end library (Table 1). Quality value (Q) is regarded to be correctly sequenced when Q20 and Q30 values are at least 90 and 85%, respectively [23]. The Q20 and Q30 values for the present study were 99.3 and 96.0%, respectively (Table 1); hence, the sequencing accuracy of *A. franciscana* was high. Additionally, the GC content of the raw data was 35.8% (Table 1).

**Table 1:** Statistics for the GSS data of *A. franciscana*

Lib ID	Raw data (bp)	Q20 (%)	Q30 (%)	GC content (%)
**PE350**	22814108862	99.3	96.0	35.8

### Genome size prediction

**Figure 1:**
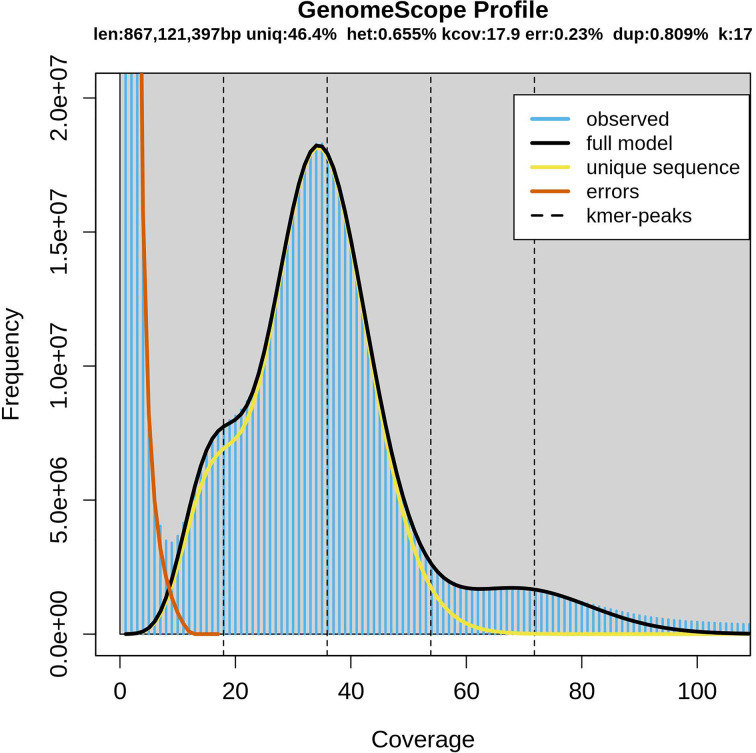
Distribution of K-mer (K = 17) Blue bars represent the observed K-mer distribution; black line represents the modeled distribution without the error K-mers (red line), up to a maximum K-mer coverage specified in the model (yellow line). Len, estimated total genome length; Uniq, unique portion of the genome (not repetitive); Het, heterozygosity rate; Kcov, mean K-mer coverage for heterozygous bases; Err, error rate; Dup, duplication rate.

In the present study, Illumina paired-end data was used for K-mer analysis using a K-value of 17. The predicted genome size was approximately 867 Mb (Figure 1). In a previous study, the genome size of *A. fransciscana* based on flow cytometry was 930 Mb [9]. Our estimation and previous results, measured using different methods, were quite similar, with a difference of 63 Mb. These estimates are smaller than 1.47 Gb in *A. salina* and 2.93 Gb in tetraploid parthenogenetic population [7,8]. In addition, the heterozygosity and duplication rates were calculated to be 0.655 and 0.809%, respectively (Figure 1).

### Genome assembly results

The results of preliminary genome assembly of *A. franciscana* are shown in Table 2. We obtained 122231 contigs with a total length of 841603395 bp. The maximum and N50 contig lengths were 1508123 and 14130 bp, respectively. The GC content of contigs was 35.50% (Table 2). Further assembly generated 46193 scaffolds with a total length of 938041450 bp. The maximum and N50 scaffold lengths were 2555521 and 67542 bp, respectively. The GC content of the scaffolds was 31.85% (Table 2).

**Table 2:** Statistics of the assembly in *A. fransciscana*

	Total length (bp)	Total number	Max length (bp)	N50 length (bp)	GC content (%)
**Contig**	841603395	122231	1508123	14130	35.50
**Scaffold**	938041450	46193	2555521	67542	31.85

These genome survey data provide useful information for genomic research of *A. franciscana* and related species. However, further study combined with more advanced NGS technologies using PacBio long read sequencing and high-throughput chromosome conformation capture (Hi-C) method are necessary to improve whole genome sequencing and assembly data. If so, *A. franciscana* could be used in a wider range of research fields (e.g. comparative genomics) as a reference genome.

### SSR loci identification

From the genome survey assembly of *A. franciscana* with a total length of ∼938 Mb, a total of 421467 repeat motifs were identified. The types of motifs contained 77.22% (325450) dinucleotide, 20.38% (85912) trinucleotide, 2.11% (8877) tetranucleotide, 0.20% (838) pentanucleotide, and 0.09% (390) hexanucleotide (Table 3). The percentage of dinucleotide repeats was the highest, and as the repeat motif length increased, the number of loci decreased, similar to other studies [13,24,25]. It has been suggested that longer repeats have higher mutation rates, causing instability [26,27] and shorter persistence times because of their downward mutation bias towards a reduction in repeat number [28].

**Table 3:** Distribution pattern of SSR motifs

Repeat motif	Number of repeats								Total
	5	6	7	8	9	10	11–20	>20	
**Dinucleotide (325450)**									
AC/GT	31132	11605	6082	3178	2092	1243	2171	244	57747
AG/CT	35678	11966	7985	5895	3480	2493	11440	7824	86761
AT/AT	79855	25592	14230	10458	6305	4932	19250	12887	173509
CG/CG	6515	796	100	22					7433
**Trinucleotide (85912)**									
AAT/ATT	21685	7466	3070	1400	510	233	1033	466	35863
ACT/AGT	17268	6614	2388	1297	385	172	586	78	28788
AAG/CTT	9019	1737	467	126	40	36	59	12	11496
AAC/GTT	3136	807	203	42					4188
ATC/GAT	1617	337	120	57					2131
AGG/CCT	1355	142	24						1521
ACC/GGT	767	139	24						930
AGC/GCT	555	51	42	15					663
CCG/CGG	176	83							259
ACG/CGT	73								73
**Tetranucleotide (8877)**									
AAAT/ATTT	1844	233	54	12					2143
AATT/AATT	720	238	12	33					1003
AATC/GATT	746	134	36			9			925
AAAG/CTTT	564	156	48	15	12		26		821
AGAT/ATCT	430	252	79	6	15	15	24		821
ACAG/CTGT	552	175	3	9		26			765
AATG/CATT	477	84	30				15		606
AAGT/ACTT	344	58	35						437
AAAC/GTTT	302	108	17						427
ACAT/ATGT	202	116	57				18	12	405
Others	380	60	66			6	12		524
**Pentanucleotide (838)**									
AATAT/ATATT	68	40	12						120
AAAAT/ATTTT	78	15	12						105
ACTAT/ATAGT	42	24	15		9		12		102
AAATT/AATTT	81	15							96
AATTC/GAATT	66								66
Others	259	57		15	6		12		349
**Hexanucleotide (390)**									
AGAGCC/GGCTCT			24	37				61	
AATATT/AATATT	15	20							35
AACAAT/ATTGTT	30								30
AATACT/AGTATT	27								27
AAATAT/ATATTT	24								24
Others	126	36		27	9		15		213
**Total**	216208	69156	35211	22631	12900	9165	34673	21523	421467

Of the dinucleotides, the most frequent motif was AT/AT (53.31%), followed by AG/CT (26.66%), AC/GT (17.74%), and CG/CG (2.28%). Of the trinucleotides, the most frequent motif was AAT/ATT (41.74%), followed by ACT/AGT (33.51%) and AAG/CTT (13.38%). ACG/CGT (0.08%) was the least frequent trinucleotides motif. The most abundant motifs among the tetra-, penta- and hexanucleotides were AAAT/ATTT (24.14%), AATAT/ATATT (14.32%) and AGAGCC/GGCTCT (15.64%), respectively (Table 3).

Overall, the motifs including A or T were more abundant than those including C or G, consistent with the findings of *Daphnia pulex* genome-wide SSR study [29]. These results might be due to the high slippage rate of A/T motifs, addition of 3′ poly(A) tail by retrotransposon elements or transition of methylated C to T residues at CpG sites [14,30–32]. These data for SSRs in *A. franciscana* will be used as valuable references for the development of microsatellite markers, although further validation studies using various *Artemia* population are needed.

## Conclusion

In the present study, the genome of *A. franciscana* was analyzed and assembled, and SSR loci were identified from the GSS data. The K-mer analysis (K = 17) estimated the genome size of *A. franciscana* to be ∼867 Mb, and the heterozygosity and duplication rates were 0.655 and 0.809%, respectively. Genome assembly results showed that contig N50 was 14130 bp, with a total length of 841603395 bp, whereas scaffold N50 was 67542 bp, with a total length of 938041450 bp. A total of 421467 SSRs were identified, of which dinucleotide motifs were the most abundant and hexanucleotide motifs were the least abundant. The present study will be a useful foundation for genomic and genetic studies on *A. franciscana*.

## Data Availability

The *A. franciscana* genome project has been registered in NCBI under the BioProject number PRJNA449186. The whole-genome sequence has been deposited in the Sequence Read Archive (SRA) database under accession numbers SRS3156165 and SAMN08892388.

## Abbreviations

GSS: genome survey sequencing
K-mer: A sequence of k characters in a string
NGS: next-generation sequencing
SSR: simple sequence repeat
